# Evaluation of glutathione S‐transferase pi 1 expression and gene promoter methylation in Moroccan patients with urothelial bladder cancer

**DOI:** 10.1002/mgg3.449

**Published:** 2018-07-24

**Authors:** Khaoula Hadami, Nadia Dakka, Mounia Bensaid, Hajar El Ahanidi, Ahmed Ameur, Hafsa Chahdi, Mohamed Oukabli, Abderrahmane Al Bouzidi, Mohammed Attaleb, Mohammed El Mzibri

**Affiliations:** ^1^ Biology and Medical Research Unit CNESTEN Rabat Morocco; ^2^ Biology of Human Pathologies Laboratory Faculty of Sciences Mohammed V University Rabat Morocco; ^3^ Military Hospital Mohammed V Rabat Morocco

**Keywords:** bladder cancer, expression, glutathione S‐transferase pi 1, Morocco, promoter methylation

## Abstract

**Background:**

Glutathione S‐transferase pi 1 (GSTP1) is a cytosolic detoxifying enzyme that protects cells against deleterious effects of oxidative stress. Deregulated expression of GSTP1 protein and aberrant promoter methylation of *GSTP1* gene were reported in various human tumors and were shown to be involved in the molecular pathway for cancer development.

**Aims and methods:**

In this study, we aimed to determine the expression status of GSTP1 in relation to its gene promoter methylation in Moroccan population of 30 bladder cancer (BC) patients and in two noncancerous bladder tissues used as controls. GSTP1 expression was assessed by immunohistochemistry and *GSTP1* gene promoter methylation status was studied by methylation‐specific PCR (MS‐PCR).

**Results:**

Glutathione S‐transferase pi 1 was expressed in the two normal tissues. In BC cases, GSTP1 expression was strong in 23.33% (7/30), moderate in 60% (18/30), and weak in 13.33% (4/30) of cases, while GSTP1 was not expressed in one cancer case (3.33%). Variability of GSTP1 expression does not correlate with high‐grade cancer or invasive‐stage (*p* > 0.05). No *GSTP1* gene promoter methylation was detected in all control and cancer cases.

**Conclusion:**

Our data suggest that GSTP1 expression is not associated with BC development, limiting its use as a biomarker for BC management in Morocco. Moreover, difference in GSTP1 expression among BC cases is not due to *GSTP1* promoter methylation.

## 1 INTRODUCTION

Bladder cancer (BC) ranks ninth in worldwide cancer incidence and affects men more likely than women (Ferlay et al., 2015). In Morocco, BC is one of the most frequently diagnosed malignancies in males. The commonest histological type is urothelial carcinoma, which occurs with either superficial or invasive phenotypes (Benider, Bendahhou, Afghar, Charrat, & Ahmadaye, 2016; Tazi, Er‐Raki, & Benjaafar, 2012). It has commonly been assumed that BC is a molecular disease arising from the multistep accumulation of many genetic and epigenetic events leading to activation of proto‐oncogenes or inactivation of tumor‐suppressor genes (Mitra, Datar, & Cote, 2006; Zhao, He, & Teng, 2016).

Glutathione S‐transferase pi 1 (GSTP1) is a cytosolic detoxifying enzyme that plays a critical role in maintaining cell integrity and protecting DNA from genotoxic and cell‐damaging molecules, it inactivates a wide variety of electrophilic carcinogens or stress‐ induced toxic intermediates by catalyzing their conjugation with reduced glutathione and making them easy for secretion (Birben, Sahiner, Sackesen, Erzurum, & Kalayci, 2012). Moreover, GSTP1 acts as a regulator of apoptosis signaling pathway through its implication in redox regulation by S‐glutathionylation of other proteins such as c‐Jun NH2‐terminal kinase (JNK; Laborde, 2010; Tew et al., 2011; Wang, Arifoglu, Ronai, & Tew, 2001). *GSTP1* gene (OMIM: *134660) is mapped to chromosome 11q13.2, spanning approximately 3 kb and comprising seven exons and six introns (Cowell, Dixon, Pemble, Ketterer, & Taylor, 1988).

In response to oxidative stress, a wide variety of stressed tumor cells show increased levels of GSTP1; GSTP1 overexpression was found in many tumors such as esophageal cancer (Joshi et al., 2005), colorectal cancer (Zhang et al., 2014), renal cancer (Kaprilian et al., 2015), lung cancer (Yang, Ebbert, Sun, & Weinshilboum, 2006), and breast cancer (Batist et al., 1986; Huang, Tan, Thiyagarajan, & Bay, 2003; Muftin, AL‐Rubaiꞌe, Yaseen, & Aziz, 2015; Vecanova et al., 2011). These studies have also demonstrated that high levels of GSTP1 correlated with cancer drugs resistance, failure of chemotherapy, and poor prognosis of tumors.

On the other hand, it was reported that loss of GSTP1 expression enhances cell susceptibility to acquire additional alterations and undergo further genetic changes toward tumor progression (Schnekenburger, Karius, & Diederich, 2014). In this field, several research works found lower GSTP1 expression in prostate cancer (Lin et al., 2001; Martignano et al., 2016; Mian et al., 2016; Zelic et al., 2016; Zhang et al., 2015), endometrial cancer (Chan et al., 2005), hepatocellular cancer (Li, Li, Gao, & Shi, 2015), and ovarian cancer (Shilpa, Bhagat, Premalata, Pallavi, & Krishnamoorthy, 2014).

It has been shown that promoter hypermethylation is an epigenetic mechanism able to repress gene transcription by inhibiting the binding of transcription factors to their consensus sequences when methylated (Herman & Baylin, 2003). In the studies cited above, downregulation of *GSTP1* gene expression was associated to an aberrant methylation in the promoter region (Chan et al., 2005; Li et al., 2015; Lin et al., 2001; Martignano et al., 2016; Mian et al., 2016; Shilpa et al., 2014; Zelic et al., 2016; Zhang et al., 2015). Moreover, promoter methylation of *GSTP1* gene was detected in some body fluids of patients with prostate cancer and was proposed as a biomarker candidate for noninvasive detection of prostate cancer (Cairns et al., 2001; Goessl et al., 2000).

Given these points, GSTP1 appears to play a driving role in multistep cancer development through a number of varying mechanisms and pathways.

The present study was planned to assess GSTP1 expression in BC biopsies and normal bladder tissues obtained from Moroccan patients and to evaluate its gene promoter methylation status in order to find a new biomarkers for better management of BC.

## 2 MATERIALS AND METHODS

### 2.1 Patients and samples preparation

Before taking part in the study, informed consent was obtained from all patients, and this study was approved by the local institutional review board (DERS/SAN/01‐16). Thirty urinary bladder biopsies were collected from Urology Department of the Military Hospital of Instruction Mohammed V in Rabat, Morocco. Each sample was divided into two similarly portions: one portion was stored at −80°C immediately after surgical removal for DNA extraction; the other portion was formalin‐fixed paraffin‐embedded (FFPE) and processed for histopathological examination in the Anatomopathology Department at the same hospital. Hematoxylin–eosin‐stained sections were graded and staged according to World Health Organization (WHO) grading criteria and Tumor Node Metastasis (TNM) staging system (Cheng, Lopez‐Beltran, & Bostwick, 2012). One noncancerous biopsy of BCG (Bacillus Calmette‐Guérin)‐treated patient and one normal urothelium tissue from malignant specimen were recruited and used as controls.

### 2.2 Imunohistochemical analysis

#### 2.2.1 Immunohistochemistry of biopsies

For immunostaining of GSTP1, FFPE biopsies were cut into 5 μm sections, deparaffinized in xylene baths and rehydrated through a series of descending ethanol baths. After that, antigen unmasking was heat‐induced using EnVision™ FLEX Target Retrieval Solution (DM828, DAKO) at 99°C for 40 min, slides were then allowed to cool at room temperature. Endogenous peroxidases were blocked with EnVision™ FLEX Peroxidase‐Blocking Reagent (SM801, DAKO) for 5 min. IHC slides were incubated with anti‐GSTP1 rabbit polyclonal antibody (ab153949, ABCAM) for 1 hr (diluted 1:500). Immunodetection was performed with EnVision™ FLEX/HRP (horseradish peroxidase) anti‐rabbit secondary antibody (SM802, DAKO) and DAB+ (diaminobenzidine) chromogenic substrate (DM827, DAKO), according to manufacturer's instructions. Slides were then counterstained with hematoxylin and dehydrated before microscopic examination.

#### 2.2.2 Analysis of GSTP1 immunohistochemical staining

Examination of IHC slides was done by light‐microscopy by histopathologists. Cells with positive GSTP1 immunostaining were brown‐stained. Two parameters were evaluated: intensity of immunostaining and percentage of positive stained tumor cells. The two parameters were separately assigned to a scoring system and were then combined in a total immunoreactive score (IRS). IRS was defined as the product of observed staining intensity (SI) and the percentage of positively stained cells (PP; Fedchenko & Reifenrath, 2014). The details of the scoring system are shown in Table 1.

**Table 1:** Details of immunoreactive scoring system (Fedchenko & Reifenrath, 2014)

SI [0–3]	PP [0–4]	IRS [SI × PP: 0–12]
No staining = 0	No stained cells = 0	0–1 = negative expression
Weak = 1	<10% = 1	2–3 = positive, weak expression
Moderate = 2	10%–50% = 2	4–8 = positive, moderate expression
Intense = 3	51%–80% = 3	9–12 = positive, strong expression
	>80% = 4	

*Note*

Staining intensity (SI) was scored from 0 to 3 and percentage of positive cells (PP) was presented in score values ranging from 0 to 4. The immunoreactive score (IRS) gives a range of 0–12 as a product of multiplication between SI score and PP score.

### 2.3 Methylation analysis

#### 2.3.1 DNA extraction

Genomic DNA was carefully extracted from the frozen section biopsies using the Isolate II Genomic DNA Kit (BIOLINE), according to manufacturer's protocol, and stored at −20°C until use. DNA concentration and purity were assayed using the NanoDrop 2000 UV‐Vis Spectrophotometer (Thermo Fisher Scientific).

#### 2.3.2 Methylation‐specific PCR

Extracted DNA was converted with sodium bisulfite using EZ DNA Methylation™ Kit (Zymo Research). Briefly, 1 μg of DNA was diluted in a final volume of 50 μl containing 45 μl of sterile water and 5 μl of dilution buffer, 100 μl of CT (Conversion Reagent) was added and the mixtures were incubated in the dark at 50°C for 12–16 hr. Modified DNA was then desulfonated using desulfonation buffer and recovered in 10 μl of elution buffer. For PCR amplification, 2 μl of bisulfite‐converted DNA was added to mixtures containing 1× PCR buffer, 1.5 mM MgCl_2_, 200 μM of each dNTP, 200 nM of each primer and 0.25 U Platinum Taq DNA polymerase (Invitrogen), in a final volume of 25 μl. PCR reactions were carried out under the following conditions: first denaturation at 95°C for 2 min, 35 cycles of 30 s at 94°C, 30 s at 60°C, 30 s at 72°C and a final extension for 7 min at 72°C. *GSTP1* gene promoter region was amplified using primers for unmethylated (U) and methylated (M) alleles (NM_000852.3), the sequences of the primers are shown in Table 2 (Chan et al., 2002). The PCR products were directly loaded onto a 2% agarose gel, stained with ethidium bromide and visualized under UV illumination. DNAs extracted from MCF‐7 and BCPAP cell lines were used as methylated and unmethylated controls, respectively.

**Table 2:** Primers used for MS‐PCR of *GSTP1* gene promoter (Chan et al., 2002)

Primer	Sequence	PCR product size
*GSTP1* U/F	5′‐GATGTTTGGGGTGTAGTGGTTGTTT‐3′	97 pb
*GSTP1* U/R	5′‐CCACCCCAATACTAAATCACAACA‐3′	
*GSTP1* M/F	5′‐TTCGGGGTGTAGCGGTCGTC‐3′	91 pb
*GSTP1* M/R	5′‐GCCCCAATACTAAATCACGACG‐3′	

*Note*

*GSTP1*: NM_000852.3.

### 2.4 Statistical analysis

The relationship between GSTP1 expression, BC clinical stage, and tumor grade was analyzed using Fisher's test. *p* values <0.05 were considered statistically significant.

## 3 RESULTS

### 3.1 Characteristics of study cases

The demographic characteristics of the 30 BC cases showed that patients’ age ranged from 42 to 78 years (mean age, 65 years), with the majority being male (*n* = 26). At clinical staging, three patients (10%) had noninvasive tumors (Ta). Among invasive cases, 19 cases had nonmuscle‐invasive disease (T1, 63%) and eight of them had invasion of the muscle layer (T2, 27%). Histological grading revealed that 12 patients had low‐grade cancer (40%) and 18 of them had high‐grade disease (60%; Table 3).

**Table 3:** Clinicopathological data of bladder cancer cases

No of patient	Age	Sex	Stage	Grade
1	56	M	Ta	LG
2	70	M	Ta	LG
3	75	F	Ta	LG
4	48	M	T1	LG
5	69	M	T1	LG
6	45	M	T1	LG
7	53	M	T1	LG
8	67	M	T1	LG
9	74	M	T1	HG
10	64	F	T1	HG
11	67	M	T1	HG
12	70	M	T1	HG
13	53	M	T1	HG
14	59	M	T1	HG
15	67	M	T1	HG
16	78	M	T1	HG
17	42	F	T1	HG
18	75	M	T1	HG
19	66	M	T1	HG
20	48	M	T1	HG
21	63	M	T1	HG
22	52	M	T1	HG
23	78	M	T2	LG
24	60	M	T2	LG
25	53	M	T2	LG
26	73	M	T2	LG
27	60	F	T2	HG
28	72	M	T2	HG
29	62	M	T2	HG
30	57	M	T2	HG
Total			Ta: 3/30 (10%)	LG 12/30 (40%)
			T1: 19/30 (63%)	
			T2: 8/30 (27%)	HG 18/30 (60%)

*Note*

F, female; HG, high grade; LG, low grade; M, male.

### 3.2 Analysis of GSTP1 expression by immunohistochemistry

**Figure 1:**
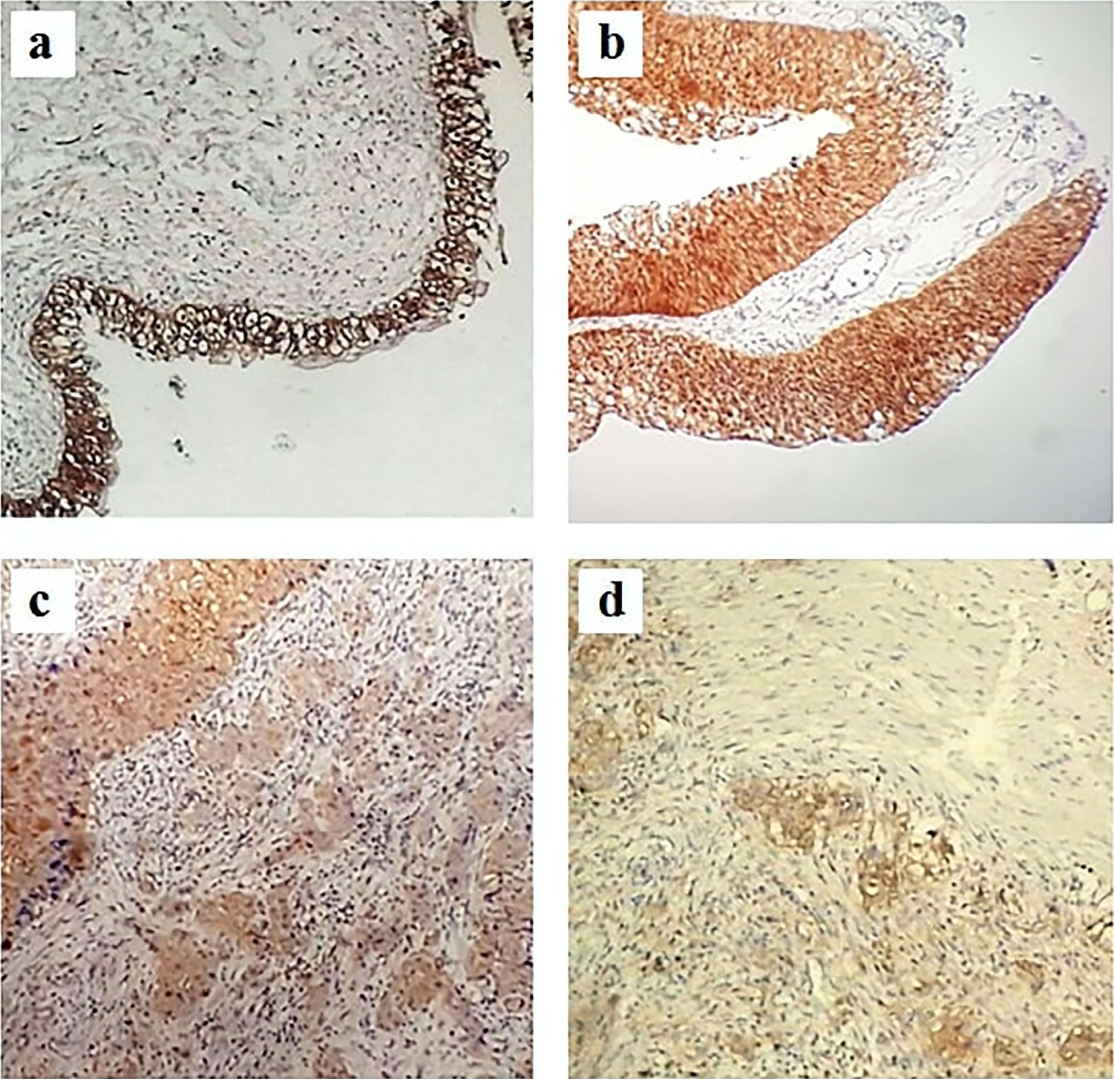
Glutathione S‐transferase pi 1 (GSTP1) immunostaining in benign bladder urothelium and different status of BC (400× magnification). (a) Benign urothelial cells showing strong immunostaining. (b) Noninvasive tumor cells with strong immunostaining. (c) Chorion‐invasive tumor cells with moderate immunostaining. (d) Muscle invasive carcinoma displaying weak immunostaining. BC, bladder cancer

Glutathione S‐transferase pi 1 expression was characterized histopathologically by brown staining and was found in both cell cytoplasm and nucleus. GSTP1 was expressed over the epithelium in benign and cancerous tissues with various GSTP1 staining intensities (Figure 1). Though, a dishomogeneous staining was observed in some areas of the tissues. GSTP1 immunoreactivity was evaluated using the IRS and results are displayed in Table 4. According to the IRS, samples were divided into groups with strong, moderate and weak GSTP1 expression.

**Table 4:** GSTP1 expression status among benign cases and bladder cancer (BC) tumors according to stage and grade

GSTP1 expression status	Controls *n* (%)	BC cases						
		Stages			*p*	Grades		*p*
		Ta *n* (%)	T1 *n* (%)	T2 *n* (%)		LG *n* (%)	HG *n* (%)	
Strong (9 ≤ IRS ≤ 12)	1 (50)	2 (66.66)	4 (21.05)	1 (12.50)	>0.05	4 (33.33)	3 (16.67)	>0.05
Moderate (4 ≤ IRS ≤ 8)	1 (50)	–	14 (73.68)	4 (50)		7 (58.33)	11 (61.11)	
Weak (2 ≤ IRS ≤ 3)	–	1 (33.33)	–	3 (37.50)		1 (8.33)	3 (16.67)	
Negative (0 ≤ IRS ≤ 1)	–	–	1 (5.26)	–		–	1 (5.55)	
*N*	2	3	19	8		12	18	
		30				30		

*Notes*

GSTP1, glutathione S‐transferase pi 1; IRS, immunoreactive score.

The *p*‐values were calculated to evaluate the association of GSTP1 expression status with tumor stage/histological grade in bladder cancer cases.

In normal tissues, GSTP1 was strongly expressed in one case and moderately expressed in the other case. Most of BC cases exhibited moderate expression (60%, 18/30), strong and weak expressions were observed in 23.33% (7/30) and 13.33% (4/30) of cases, respectively. GSTP1 was not expressed in one cancer case (3.33%).

In terms of clinical stages, strong GSTP1 expression was found in 66.66% (2/3) of noninvasive tumors (Ta), in 21.05% (4/19) of nonmuscle‐invasive tissues (T1) and in 12.50% (1/8) of muscle‐infiltrating tumors (T2). Moderate expression was observed in 73.68% (14/19) of stage T1 and in 50% (4/8) of stage T2. Weak expression has appeared in 33.33% (1/3) of stage Ta and in 37.50% (3/8) of stage T2. Negative GSTP1 expression was observed in 5.26% (1/19) of T1 stage. Statistically, no significant association was obtained between GSTP1 expression and BC invasion (*p *> 0.05).

Among samples with low‐grade cancer, 33.33% of cases (4/12) exhibited strong GSTP1 expression, 58.33% of them (7/12) showed moderate expression, while 8.33% (1/12) had weak GSTP1 expression. In high‐grade samples, 16.67% of cases (3/18) showed strong GSTP1 immunoreactivity, 61.11% of samples (11/18) displayed moderate GSTP1 staining, 16.67% had weak staining (3/18) and 5.55% (1/18) showed none staining. Variability of GSTP1 expression did not correlate with cell transition from low‐ to high‐grade (*p *> 0.05).

### 3.3 *GSTP1* gene promoter methylation

In MCF‐7 cell line, used as methylation positive control, both unmethylated and methylated alleles were detected; while only unmethylated alleles were amplified in BCPAP cell line that was used as methylation negative control.

**Figure 2:**
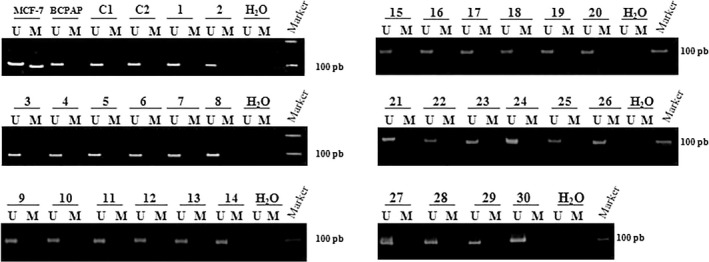
Results of methylation‐specific PCR (MS‐PCR) for *GSTP1* promoter region. Lanes U and M correspond to amplification with primers recognizing unmethylated (97 bp) and methylated (91 bp) sequences, respectively. DNA from MCF‐7 cell line was used as positive control for methylation and DNA from BCPAP cell line was used as negative control. Results of normal bladder urotheliums (C1, C2: Control 1, 2) and the 30 BC cases (1–30) are represented. Water was used as negative PCR control (H_2_O). On the right side: the 100 bp DNA ladder. *GSTP1*: NM_000852.3. BC, bladder cancer

*GSTP1* promoter appears unmethylated in all BC and nonmalignant tissues. Indeed, none of the benign samples and the 30 cancer cases (all stages and grades mingled) showed methylated alleles, only the unmethylated alleles were detected (Figure 2).

## 4 DISCUSSION

Given its antioxidative and detoxification capabilities, GSTP1 protein was found to be expressed in most normal human tissues with a predominance in epithelial cells of respiratory, digestive and urinary tracts that are the most exposed systems to carcinogens (Savic‐Radojevic et al., 2007; Terrier, Townsend, Coindre, Triche, & Cowan, 1990). In comparison with normal tissues, variability of GSTP1 levels was described in different human cancers.

Some studies indicated an overexpression of *GSTP1* gene in esophageal cancer (Joshi et al., 2005), colorectal cancer (Zhang et al., 2014), renal cancer (Kaprilian et al., 2015), lung cancer (Yang et al., 2006), and in BC (Chen et al., 2013; Pljesa‐Ercegovac et al., 2011; Savic‐Radojevic et al., 2007). Other studies showed reduced *GSTP1* gene expression in prostate, endometrial, hepatocellular, and ovarian cancers (Chan et al., 2005; Li et al., 2015; Lin et al., 2001; Martignano et al., 2016; Mian et al., 2016; Shilpa et al., 2014; Zelic et al., 2016; Zhang et al., 2015). The results were controversial in breast cancer, a number of studies found overexpressed *GSTP1* gene (Batist et al., 1986; Huang et al., 2003; Muftin et al., 2015; Vecanova et al., 2011), while others reported underexpressed *GSTP1* gene (Esteller et al., 1998; Fang et al., 2015).

In this study, we evaluated the expression of GSTP1 by IHC in 30 BC biopsies and two noncancerous bladder tissues obtained from Moroccan patients. As expected, we detected GSTP1 protein mainly in cell cytoplasm; still, nucleic staining was also observed. The nuclear localization of GSTP1 has been described previously (Ali‐Osman, Brunner, Kutluk, & Hess, 1997; Huang et al., 2003; Pljesa‐Ercegovac et al., 2011), its accumulation into the nucleus was ascribed to the overexpressed Bcl‐2 protein that has been implicated as the regulator of transport of GSTP1 through the nuclear pore complex (Pljesa‐Ercegovac et al., 2011). During IHC examination, dishomogeneous GSTP1 staining was observed in most of the tissues. This heterogeneous staining was also reported in the prostate (Martignano et al., 2016).

Our immunohistochemical analysis demonstrated that GSTP1 is expressed in normal samples, showing strong or moderate expression and in most of the tumor cases, in which the expression was varying between strong, moderate, and weak. However, variant GSTP1 expression did not significantly correlate with the progression or malignant behavior of cancer (*p *> 0.05). Also, an interindividual variability of GSTP1 expression in the normal samples and among patients with the same stage or grade of cancer was observed.

In comparison to our findings, several studies have reported an upregulated GSTP1 expression which increased gradually with high‐grade tumor or invasive‐stage and was linked to decreased levels of urothelial cells apoptosis (Chen et al., 2013; Pljesa‐Ercegovac et al., 2011; Savic‐Radojevic et al., 2007).

Because of the differential expression of GSTP1 in our specimens, we analyzed the methylation status of *GSTP1* gene promoter region by MS‐PCR to verify its possible association with underexpressed GSTP1 cases. As a result, none of the normal bladder tissues or tumor samples expressed any methylated alleles. The methylation status of the promoter region of *GSTP1* gene is controversial. In some studies, *GSTP1* was found to be infrequently methylated and was not associated with grading or muscle invasiveness of urinary BC (Chan et al., 2002; Hauser et al., 2013; Maruyama et al., 2001; Yates et al., 2006). However, Sacristan et al. found that *GSTP1* promoter was methylated in 44.6% of cases, but the methylation decreased with cancer progression, it classified Ta versus T1 stages and distinguished LG versus HG tumors (Sacristan et al., 2014). In addition, Casadio et al., reported that *GSTP1* methylation is capable of significantly predicting nonmuscle‐invasive BC recurrence, its methylation frequencies were higher in nonrecurring than recurring tumors (26% vs. 5%) and were significantly indicative of a lack of recurrence at the 5‐year follow‐up (*p *=* *0.02) (Casadio et al., 2013). All findings considered, the role of GSTP1 in the carcinogenesis of the bladder has yet to be defined.

Our finding of reduced GSTP1 expression in tumors not displaying methylated alleles was also reported in a study of Shilpa et al. (2014) suggesting that promoter methylation may not be the only regulator mechanism in gene silencing; hence, other mechanisms may occur and affect GSTP1 transcription. Actually, transcription factors such as SP1, AP‐1, NF‐κB, and GATA1 were reported to play an important role in the regulation of GSTP1 expression (Schnekenburger et al., 2014). Similarly, Lo et al. (2008) showed the ability of wild‐type p53 to transcriptionally activate *GSTP1* gene, in that case, low GSTP1 protein level was associated with mutant p53. In the same way, Uchida et al. (2013) demonstrated that GSTP1 expression may be repressed epigenetically by several miRNAs notably the miR‐133a. Moreover, it should be noted that there is an individual variation of GST‐Pi expression related to dietary and lifestyle factors (Coles & Kadlubar, 2003).

Overall, this study is very informative: (a) GSTP1 is expressed in normal bladder tissues and in the majority of BC cases, showing a variation from intense to weak GSTP1 immunoreactivity; (b) the protein expression is not associated with disease’ stage or grade; (c) GSTP1 down‐expression found in some samples was not caused by gene promoter methylation. However, our study has some limitations, mainly the small sample sizes of both BC cases and normal controls making difficult to draw concise conclusions.

In conclusion, this preliminary study showed that GSTP1 expression is not associated with BC development and therefore, GSTP1 expression could not be used as a biomarker for BC management in Morocco. Moreover, difference in GSTP1 expression among BC cases is not due to *GSTP1* promoter methylation.

## CONFLICT OF INTEREST

None declared.
